# Efficacy and safety of fire needle therapy in treating non‐segmental stable vitiligo: A randomized self‐controlled clinical trial

**DOI:** 10.1111/jocd.16428

**Published:** 2024-06-18

**Authors:** Yuyi Wang, Mao Lin, Fang Huang, Ruiling Jia, Wei Xu, Qi He, Qiong Xiong, Qin Hu, Qingchun Diao, Zhaolan Liu

**Affiliations:** ^1^ Centre for Evidence Based Chinese Medicine Beijing University of Chinese Medicine Beijing China; ^2^ Department of Dermatology Chongqing Hospital of Traditional Chinese Medicine Chongqing China; ^3^ Department of Dermatology, West China Hospital Sichuan University Chengdu Sichuan China; ^4^ Chongqing Jiulongpo District Shiqiaopu Community Health Service Center Chongqing China

**Keywords:** fire needle therapy, randomized controlled trial, repigmentation, tacrolimus, vitiligo

## Abstract

**Background:**

Vitiligo remains a challenging condition to treat. Fire needle therapy, a traditional Chinese medicine technique, has potential as an alternative therapeutic strategy. However, rigorous evidence on its efficacy is lacking.

**Objective:**

We aimed to evaluate the efficacy and safety of fire needle therapy, alone and combined with topical tacrolimus ointment, for non‐segmental stable vitiligo.

**Methods:**

In this 6‐month randomized self‐controlled trial, 35 vitiligo patients were enrolled, providing three similar lesions each. Lesions were randomly allocated to receive fire needle monotherapy, 0.1% tacrolimus ointment monotherapy, or combined fire needle and tacrolimus ointment therapy. The main outcome was change in vitiligo surface area.

**Results:**

In total, 29 patients completed the 6‐month follow‐up. The combination therapy group showed significantly greater reductions in vitiligo surface area compared to monotherapy groups starting at months 4 and 5. By the end of the study, combination therapy resulted in remarkably higher repigmentation responses, with 89.7% of lesions showing at least mild (≥25%) repigmentation and 51.7% showing good (≥50%) repigmentation. This significantly exceeded the outcomes with topical tacrolimus ointment alone, which only achieved 6.9% mild response and 6.9% good response. Fire needle monotherapy also demonstrated steady repigmentation over time, with 69% of lesions attaining a mild response by month 6. Importantly, no major adverse events occurred.

**Conclusion:**

This study provides promising preliminary evidence supporting the use of fire needle therapy, alone or in combination with topical tacrolimus ointment, for inducing repigmentation in non‐segmental stable vitiligo. As a non‐pharmacological approach, fire needle therapy warrants further study as an alternative vitiligo treatment.

## 1 BACKGROUND

Vitiligo is a chronic autoimmune disorder characterized by the selective destruction of melanocytes, resulting in the depigmentation of skin and hair. This condition affects between 0.5% and 2.0% of the global population, with no significant differences in the prevalence based on sex or race.^1^, ^2^ Patients with vitiligo typically present with white macules and patches on their skin and hair, which tend to enlarge and spread over time.^1^, ^3^ Despite extensive research, the exact pathogenesis of vitiligo remains elusive. However, several theories have been proposed to explain the underlying mechanisms that lead to the destruction of melanocytes. These theories implicate various factors, including immune function, oxidative stress, melanocyte self‐destruction, genetics, and neurochemical factors, among others.^3^, ^4^, ^5^ Current understanding suggests that vitiligo is a multifactorial disease, wherein both environmental and genetic factors contribute to its onset.^2^, ^3^, ^6^

An international consensus meeting identified two types of vitiligo: non‐segmental vitiligo (NSV) and segmental vitiligo.^7^ NSV, which is characterized by symmetric body distribution, accounts for 85%–90% of cases.^8^ Vitiligo is more than just a “cosmetic condition”^9^; it profoundly impacts patients' self‐esteem and induces significant psychological distress, especially for those with lesions on exposed areas such as hands and face.^10^, ^11^, ^12^ Studies have shown that vitiligo's effects on quality of life and psychological impairment are comparable to those of other skin diseases like psoriasis and atopic dermatitis.^10^ Social stigma and misconceptions surrounding vitiligo continue to affect patients adversely.^2^ Therefore, the timely provision of proper treatment is critical for supporting patients both physically and psychologically.

At present, vitiligo remains incurable, yet numerous therapeutic approaches exist that aim to promote the regeneration of skin pigment.^1^, ^3^, ^4^ Common treatment methods include the use of topical corticosteroids, calcineurin inhibitors, phototherapy, and surgical interventions.^2^, ^7^, ^13^, ^14^, ^15^, ^16^ Despite the availability of these options, treating vitiligo continues to be one of the most challenging tasks in dermatology. The effectiveness of these treatments is somewhat limited, and their success in achieving repigmentation is contingent upon several factors, such as the course of the disease, lesion location, and severity of the condition. Additionally, these treatments can lead to several adverse effects, such as skin atrophy, hyperpigmentation, and even skin cancer in severe cases.^15^, ^16^ As a result, there is a pressing need for the inception of innovative treatments.

In recent years, traditional Chinese medicine (TCM) has garnered increased interest in vitiligo treatment, with fire needle therapy emerging as a promising alternative.^17^, ^18^, ^19^, ^20^, ^21^ Fire needle therapy, a form of traditional Chinese acupuncture, boasts a history spanning thousands of years. This technique entails heating needles before rapidly inserting them into affected white patches. Several case series studies have reported the efficacy of fire needle therapy in fostering repigmentation and enhancing the overall appearance of vitiligo lesions.^17^, ^18^ Nonetheless, well‐designed randomized controlled trials are critically needed to corroborate these therapeutic benefits. Accordingly, we conducted a randomized, self‐controlled study to investigate the efficacy and safety of fire needle therapy in treating non‐segmental stable vitiligo.

## 2 METHODS

### 2.1 Study design

This randomized, self‐controlled study was conducted from July 2020 and February 2023 at the Department of Dermatology, Chongqing Hospital of Traditional Chinese Medicine. All research procedures adhered to the principles of the Helsinki Declaration. The final protocol and informed consent documentation received approval from the ethics committee of Chongqing Hospital of Traditional Chinese Medicine (2020‐ky‐24).

### 2.2 Participants

Patients with vitiligo seeking initial diagnosis or follow‐up at the dermatology clinic of Chongqing Hospital of Traditional Chinese Medicine were recruited. The diagnosis was confirmed by experienced dermatologists according to the "Chinese Consensus on the Diagnosis and Treatment of Vitiligo (2018 edition)."^22^ Written informed consent was obtained from all participants before enrollment.

The inclusion criteria were as follows: (1) clinical diagnosis of NSV^7^; (2) stable white patches for over 6 months; (3) a minimum of three adjacent or symmetrical lesions, or one large lesion dividable into two or three lesions; (4) involvement of less than 10% body surface area (BSA); and (5) age between 18 and 65 years old.

Exclusion criteria included: (1) segmental vitiligo; (2) lesions on hands or feet; (3) any treatments potentially affecting vitiligo within specified timeframes, such as systemic treatment within 8 weeks, phototherapy within 4 weeks, or topical treatment within 2 weeks; (4) pregnancy or breastfeeding; and (5) high risk of keloid formation.

### 2.3 Sample size calculation

Sample size calculation was performed using PASS 15 software. Based on a pilot trial, the percentage of patients achieving 50% repigmentation was 60% in the combination therapy group, 50% in the fire needle therapy group, and 20% in the topical tacrolimus ointment group. A sample size of 82 lesions was determined to provide 80% power to detect an effect size of 0.3430 using a chi‐square test with 2 degrees of freedom and a significance level (*α*) of 0.05. Accounting for a 20% dropout rate, a total of 103 skin lesions were required, originating from 35 patients with vitiligo, with 3 lesions per patient.

### 2.4 Randomization and blinding

Each patient selected either three adjacent or symmetrical white patches, two patches (by dividing a larger lesion), or one larger patch separated into three sections. The selected patches were numbered sequentially from left to right and top to bottom. Using a random block generated by a SAS program and sequentially numbered opaque envelopes, the three patches were randomly allocated to receive either single‐use fire needle therapy, single‐use tacrolimus ointment, or a combination of fire needle therapy and tacrolimus ointment. Two blinded dermatologists evaluated the outcomes monthly.

### 2.5 Treatment

#### 2.5.1 Fire needle therapy group

One hour before treatment, the white patches were anesthetized with lidocaine ointment (produced by Tongfang Pharmaceutical Group Co., Ltd.). Patients were positioned comfortably, and the patches were disinfected. The acupuncturist held 3–5 needles (0.34 mm × 25 mm, Suzhou Medical Supplies Factory Co., Ltd.) simultaneously and heated the tips until red using an alcohol lamp (Figure 1). The needles were rapidly inserted into the patches and swiftly removed at a frequency of 2–3 times per second, with a 2–3 mm puncture interval and a 1 mm depth (Figure 1). To prevent infection, patients were instructed to avoid water contact with treated lesions for 24 h. Treatment was administered biweekly for 6 months (Figure 2).

**FIGURE 1:**
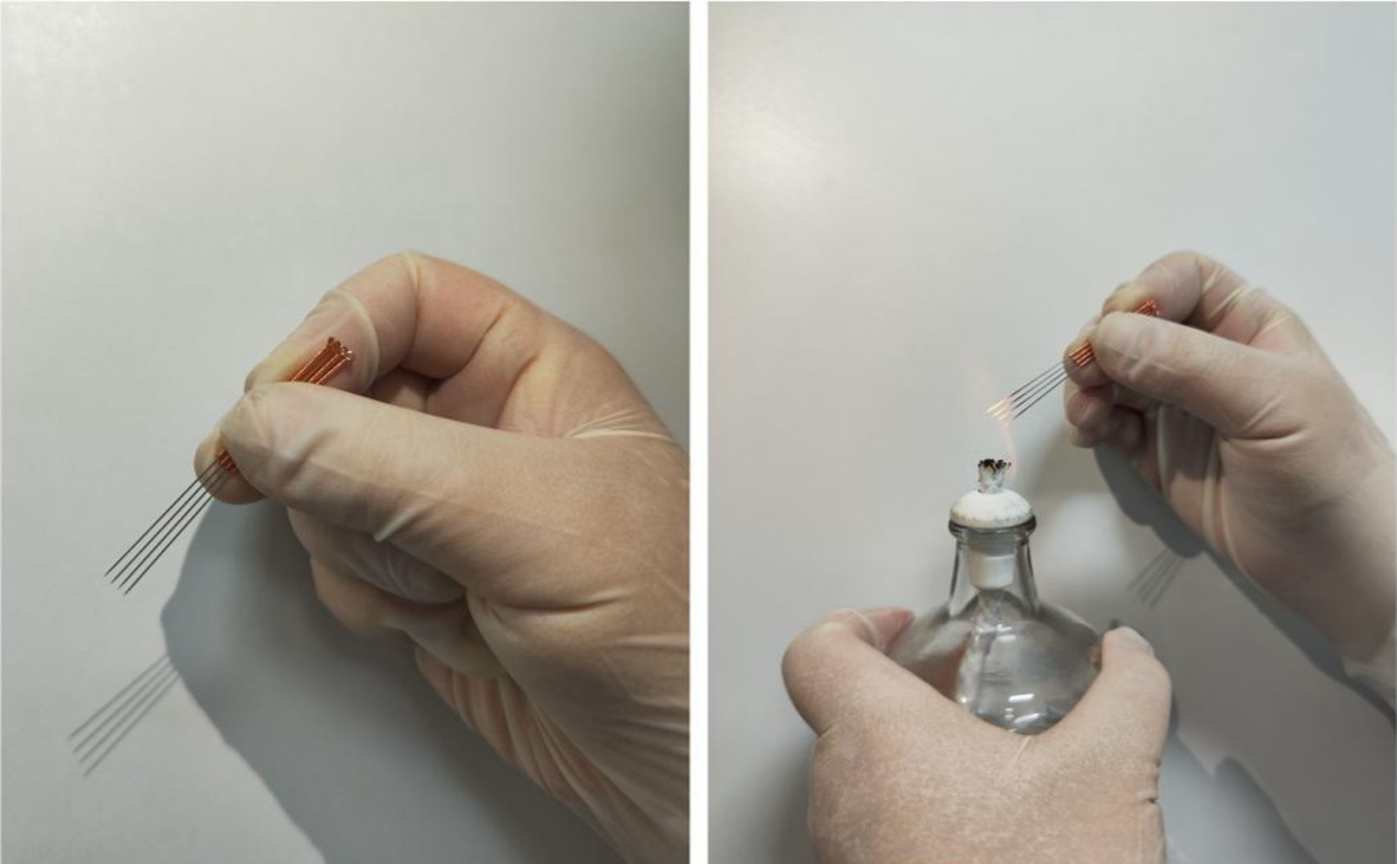
The filiform needles (0.34 mm × 25 mm) used in fire needle therapy for vitiligo treatment.

**FIGURE 2:**
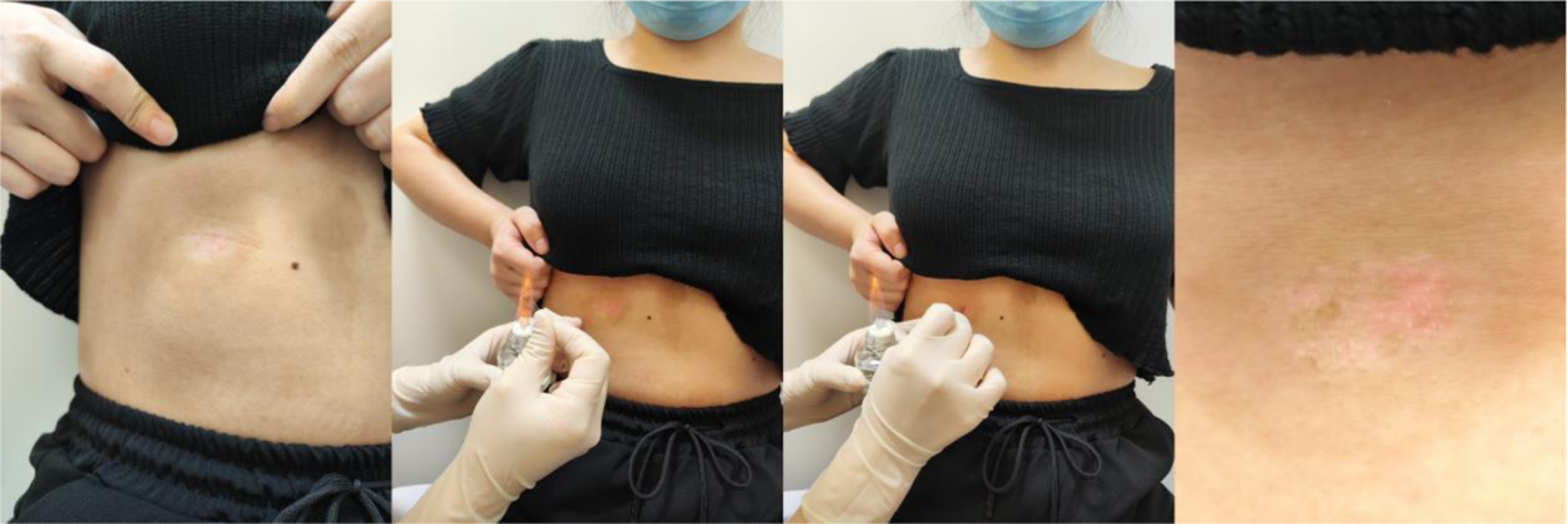
Procedure for the treatment of vitiligo with fire needles.

#### 2.5.2 Topical tacrolimus ointment group

Patients applied 0.1% tacrolimus ointment (Astellas Pharma China Co., Ltd.) to the affected patches twice daily for 6 months.

#### 2.5.3 Combination therapy group

Patients received both fire needle therapy and topical tacrolimus ointment. Fire needle therapy was administered biweekly, while 0.1% tacrolimus ointment was applied twice daily for 6 months, following the same regimens as described above. On days when fire needle therapy was performed, patients refrained from using tacrolimus ointment.

We provided each patient with a diary card and instructed them to record the time and location of their tacrolimus application, thereby improving patient compliance with the use of tacrolimus.

### 2.6 Outcomes

#### 2.6.1 Primary outcome

Vitiligo surface area: Vitiligo surface area was evaluated using the grid counting method^23^ (Figure 3). This method involves covering the white patch with a transparent plastic sheet and outlining the patch shape with a marker. As shown in Figure 3, the distance between two dots on the plastic sheet is 0.33 cm, and the area of a grid consisting of four dots is 0.1 cm^2^. To estimate the total lesion area, the number of grids is multiplied by 0.1 cm^2^. Grids covering more than half of the lesion are included in the count, while those covering less than half are disregarded.

**FIGURE 3:**
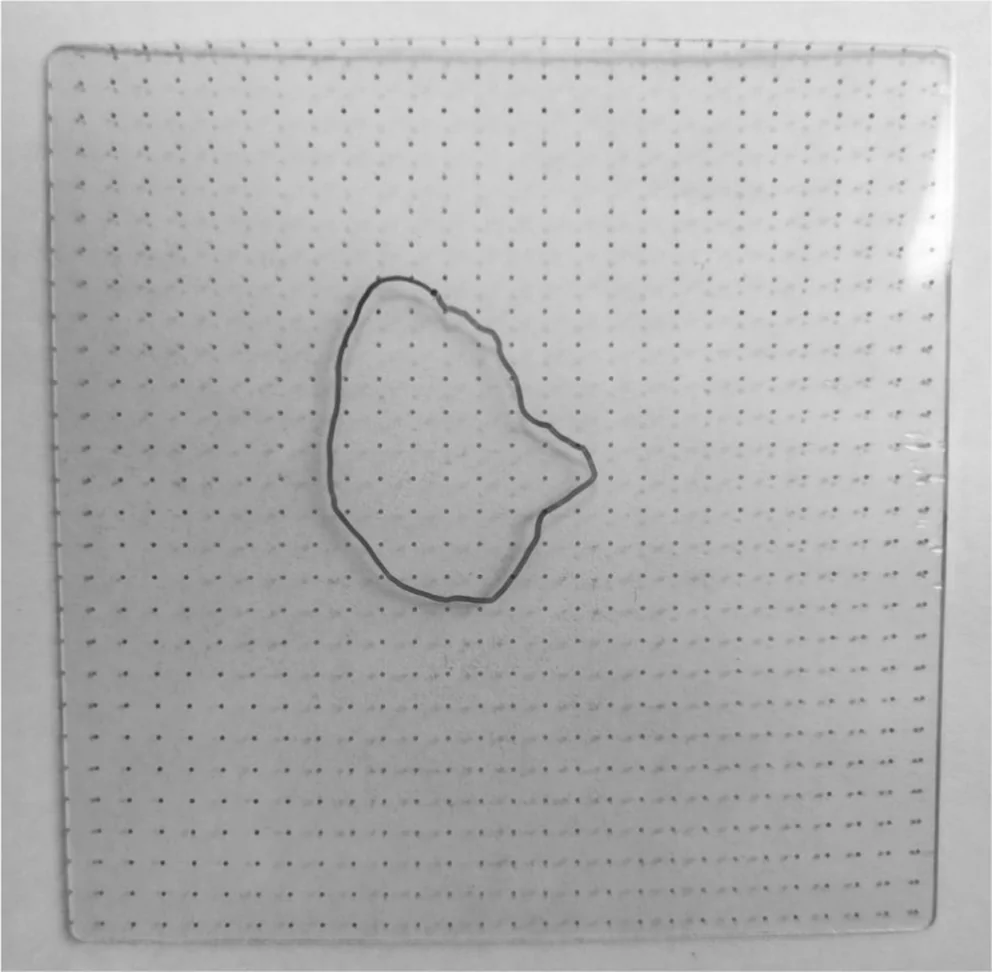
The transparent plastic sheet for the evaluation of vitiligo surface area.

#### 2.6.2 Secondary outcomes

Mild response: the percentage of patients achieving ≥25% repigmentation.

Good response: the percentage of patients achieving ≥50% repigmentation.

In the evaluation of the degree of repigmentation, we referred to the Vitiligo Area Scoring Index (VASI) classification for the degree of depigmentation.^24^ We categorized the degree of repigmentation into 25% and 50% repigmentation (as shown in Figure 4).

**FIGURE 4:**
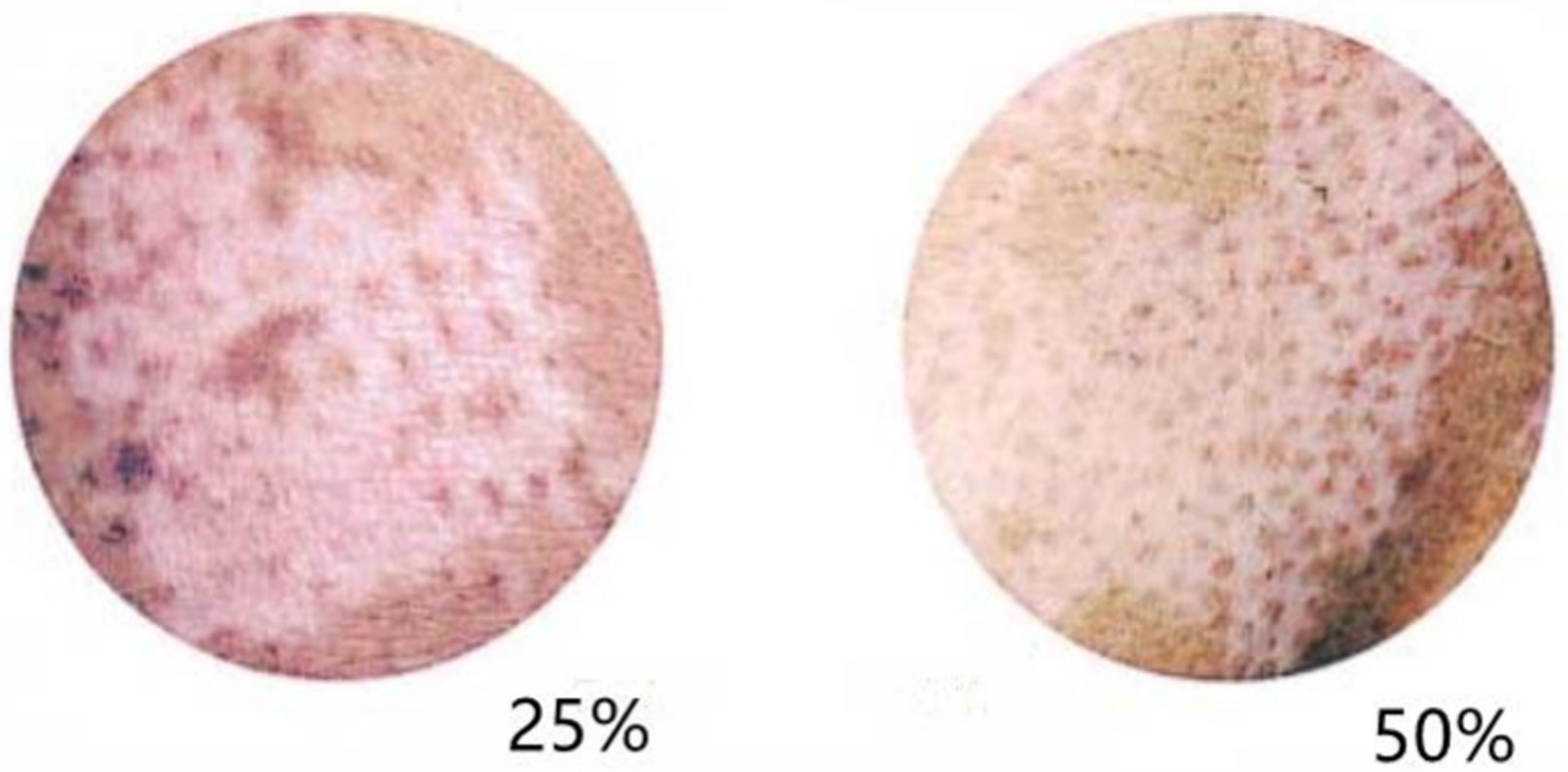
Criteria for the degree of repigmentation.

#### 2.6.3 Safety outcomes

Adverse events (AEs) were closely monitored during each monthly visit throughout the study to ensure patient safety.

### 2.7 Statistical analysis

Quantitative data that follow a normal distribution were expressed as the mean ± standard deviation. Quantitative data that do not follow a normal distribution were expressed as the median and interquartile range (25th, 75th percentile). Categorical data were shown as numbers and percentages. The primary outcome was analyzed using a generalized linear mixed model. Further pairwise comparisons across time points and groups were conducted using the Friedman test. Secondary outcomes were analyzed using the chi‐square test. Pairwise comparisons between the groups were adjusted using the Bonferroni method. The dataset used for statistical analysis was the per‐protocol population. All statistical analyses were performed using a SPSS Statistics version 22.0 (SPSS, Inc., Chicago, IL, USA), and a *p* < 0.05 was considered statistically significant for all tests.

## 3 RESULTS

### 3.1 Patient recruitment and characteristics

A total of 76 patients were screened for participation in this study, of which 35 patients, accounting for 105 lesions, met the inclusion criteria. The study population consisted of 16 males and 19 females, with a mean age of 37.3 ± 12.6 (SD) years (range: 18–63 years). The mean disease duration was 7.9 ± 5.5 (SD) years. All included patients had white patches distributed on the trunk and extremities, with no facial involvement. Comorbidities, including atopic dermatitis, alopecia areata, and others, were present in 25.7% of the patients. Six patients withdrew from the study due to needling pain (*n* = 1), busy work schedules (*n* = 2), and the impact of the COVID‐19 pandemic (*n* = 3). A total of 29 patients completed the 6‐month follow‐up. The screening process and patient characteristics are detailed in Figure 5 and Table 1.

**FIGURE 5:**
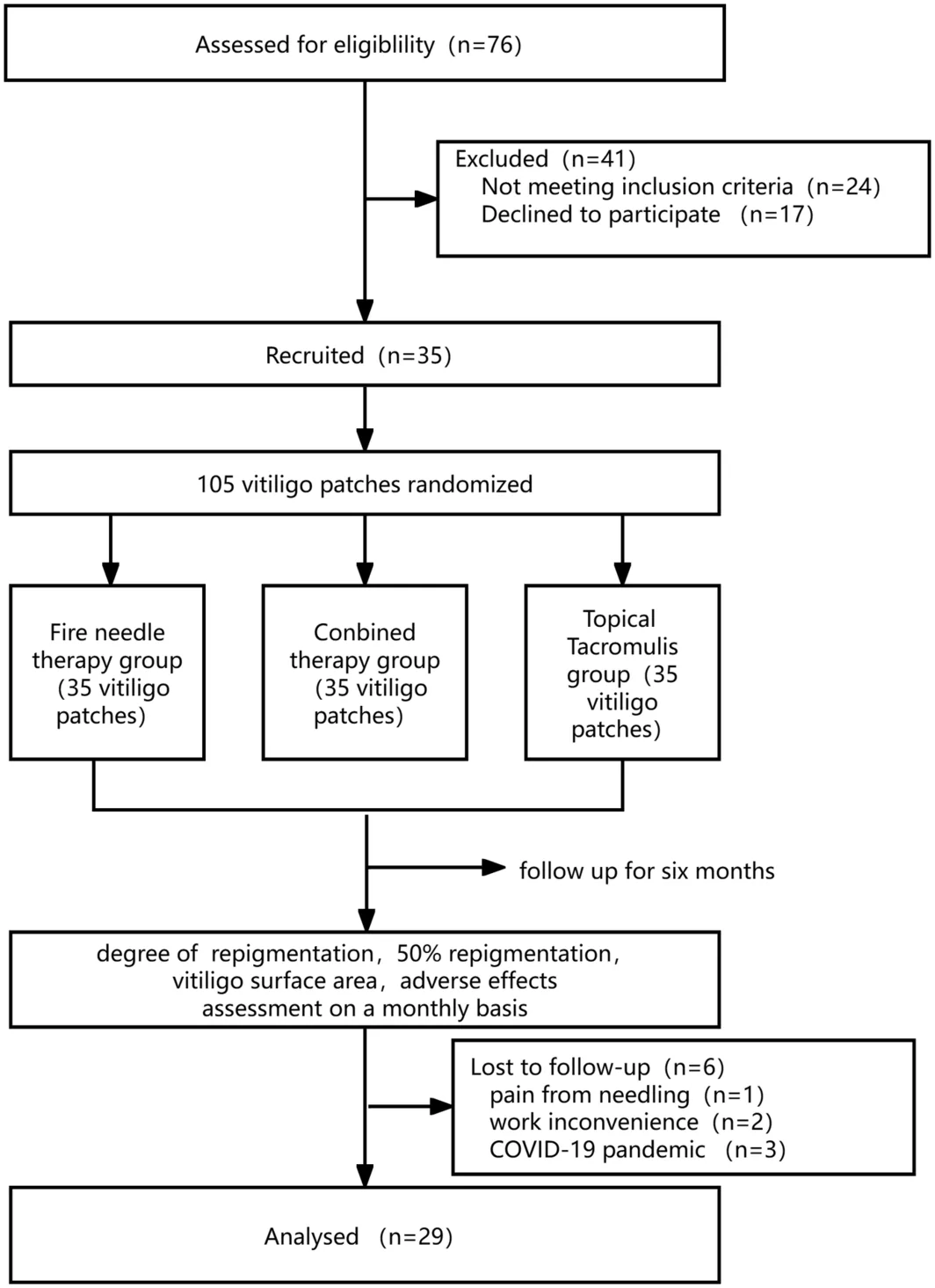
Flow diagram of patient enrollment, randomization and analysis.

**TABLE 1:** Baseline characteristics of included patients (*n* = 35).

No.	Gender	Age	Disease duration (years)	Location	Hair involvement	Family history	Comorbidities	Vitiligo surface area (mm^2^)			Completed/withdrew
								Group A	Group B	Group C	
1	Female	63	3	Trunk	No	No	Allergic rhinitis	25.6	22.3	19.4	Completed
2	Male	37	9	Trunk	No	No	Hyperuricemia	7.3	7.6	3.9	Completed
3	Male	24	5	Trunk	No	No	No	2.2	4.2	3.3	Completed
4	Male	34	7	Trunk	No	No	No	2.9	2.8	2.7	Completed
5	Male	33	3	Trunk	No	No	Fatty liver	11.7	13.6	17	Completed
6	Female	32	4	Trunk	No	No	No	3.4	3.1	6.7	Completed
7	Male	21	10	Trunk	No	No	No	15.7	16.1	15.6	Completed
8	Male	35	3	Trunk	No	No	No	8.2	7.2	8.9	Completed
9	Female	19	8	Trunk	No	No	No	1.2	1	4.1	Completed
10	Male	31	9	Trunk	No	No	No	14.6	10.9	14.5	Completed
11	Male	30	1	Arm	No	No	No	1.3	1.6	0.9	Completed
12	Female	33	3	Neck	Yes	Grandpa	Allergic purpura	6.7	7.9	5.4	Completed
13	Male	21	3	Trunk	No	No	Alopecia areata	1.6	2.2	1	Completed
14	Female	43	13	Trunk	No	No	No	4.2	3.7	3.8	Completed
15	Female	52	16	Neck	No	Grandma	No	13.2	11.3	10.5	Withdrew
16	Male	57	17	Neck	No	No	Diabetes	7.8	5.6	6.5	Withdrew
17	Female	43	12	Neck	No	Sister	No	11.2	5.7	4.5	Completed
18	Female	58	4	Neck	No	No	Hypertension	3.8	3.5	3.7	Completed
19	Male	25	2	Trunk	No	No	No	2.4	4.5	3.4	Withdrew
20	Male	45	10	Trunk	No	No	No	4.5	6.5	5.6	Withdrew
21	Female	53	4	Trunk	No	No	No	3.5	3	3.2	Completed
22	Male	42	27	Neck	No	No	Alopecia areata	5.6	4.6	5.4	Withdrew
23	Female	60	15	Neck	No	No	No	1.2	1.5	1.1	Completed
24	Female	18	8	Neck	No	No	No	2.8	2.6	2.4	Completed
25	Female	39	3	Trunk	No	No	No	2.2	4.7	4.6	Withdrew
26	Male	20	4	Trunk	No	No	No	6.9	4.3	3.2	Completed
27	Male	37	5	Trunk	No	No	No	3.4	4.6	6.4	Completed
28	Female	26	4	Trunk	No	No	Atopic dermatitis	7.6	5.6	6.4	Completed
29	Female	45	3	Trunk	No	No	No	2.7	1.4	2.2	Completed
30	Male	52	6	Trunk	No	No	No	3.6	3.4	3.8	Completed
31	Male	33	11	Trunk	No	No	No	2.4	3.2	2.7	Completed
32	Male	38	3	Thigh	No	No	No	3.2	2.8	2.6	Completed
33	Female	35	6	Trunk	No	No	No	4.3	3.4	5.2	Completed
34	Female	47	2	Trunk	No	No	No	1.5	2.3	1.4	Completed
35	Male	23	5	Trunk	No	No	No	25.6	22.3	19.4	Completed

### 3.2 Vitiligo surface area

Figure 6 shows the changes in median vitiligo surface area over time for the three groups. As the data did not conform to a normal distribution and the groups were not independent, a generalized linear mixed‐effects model was used. After correcting for baseline area, the results showed significant differences in vitiligo surface area between the groups and at different time points (*p* < 0.05) (Table 2). The Friedman test demonstrated that starting from the fourth month, the combination therapy group exhibited a greater reduction in vitiligo surface area compared to the topical tacrolimus ointment group (*p* = 0.018). From the fifth month onward, the combination therapy group outperformed the fire needle therapy group (*p* < 0.001). However, there were no statistically significant differences between the fire needle therapy and topical tacrolimus ointment groups (*p* > 0.05). Compared to pretreatment, the fire needle, combination, and topical tacrolimus groups showed statistically significant differences starting at months 3, 2, and 5, respectively (*p* < 0.001).

**FIGURE 6:**
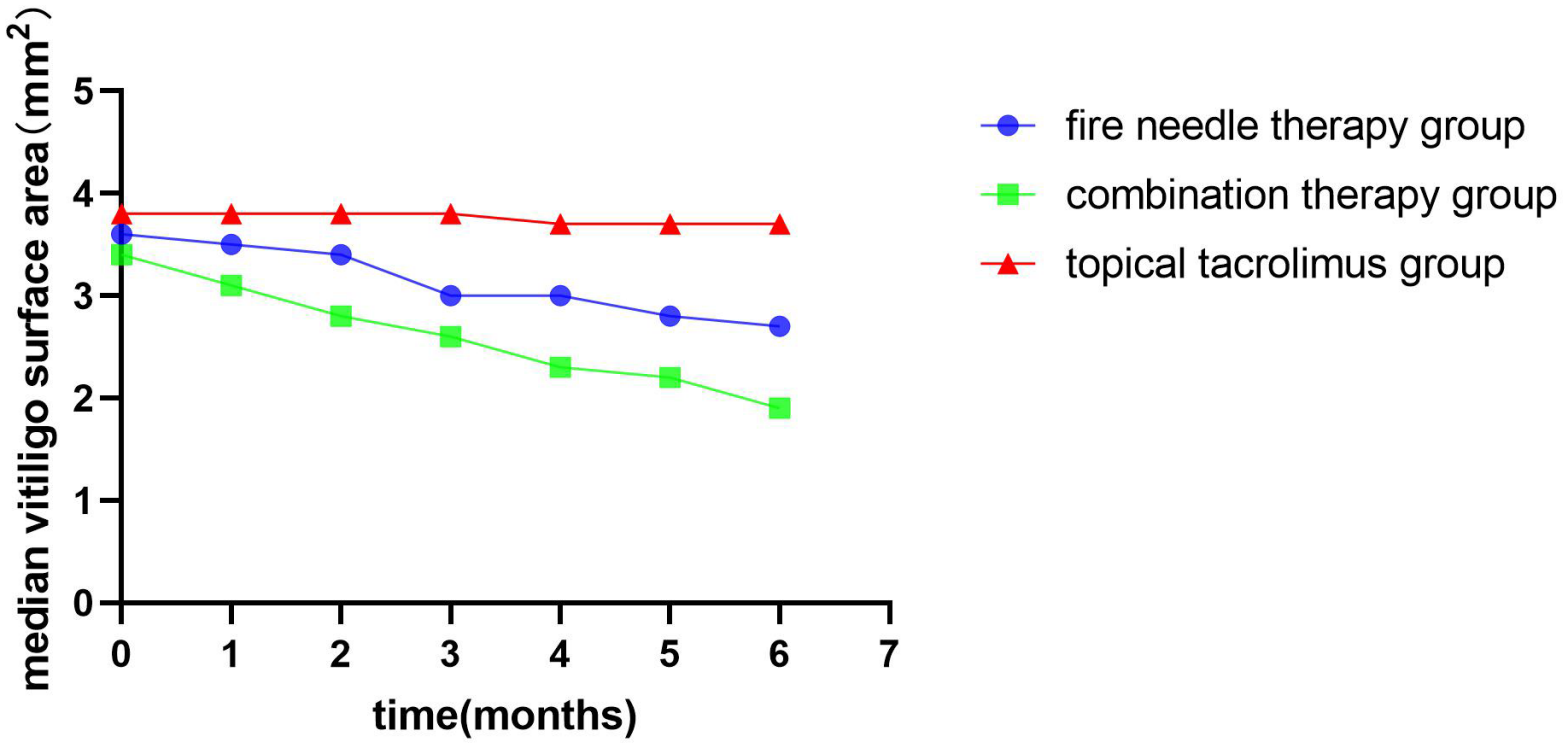
Changes in median vitiligo surface area over time for three groups.

**TABLE 2:** Median vitiligo surface area at different time points for three groups (*n* = 29).

Group	Baseline (IQR, mm^2^)	One month (IQR, mm^2^)	Two months (IQR, mm^2^)	Three months (IQR, mm^2^)	Four months (IQR, mm^2^)	Five months (IQR, mm^2^)	Six months (IQR, mm^2^)	*X* ^2^ *	*p*
Group A	3.60 (2.55,7.45)^a^	3.50 (2.50,6.95)^a^	3.40 (2.40,6.70)^ac^	3.00 (2.10,6.50)^bc^	3.00 (1.90,6.40)^bAB^	2.80 (1.70,6.00)^bdA^	2.70 (1.45,5.60)^dA^	159.28	<0.001
Group B	3.40 (2.70,6.45)^a^	3.10 (2.50,5.50)a	2.80 (2.00,5.40)^ab^	2.60 (1.75,5.25)^bc^	2.30 (1.55,5.05)^cdA^	2.20 (1.30,4.10)^deB^	1.90 (0.85,3.55)^eB^	163.30	<0.001
Group C	3.80 (2.65,6.40)^a^	3.80 (2.50,6.40)^a^	3.80 (2.50,6.35)^a^	3.80 (2.45,6.35)ac	3.70 (2.40,6.35)^abcB^	3.70 (2.40,6.10)^bcA^	3.70 (2.30,6.05)^bA^	104.50	<0.001
*X* ^2^ *	4.35	2.97	1.81	4.84	8.09	17.33	27.49		
*p*	0.114	0.226	0.405	0.089	0.018	<0.001	<0.001		

*Note*: Group A, fire needle therapy; Group B, combination therapy; Group C, topical tacrolimus ointment; IQR, median and interquartile range.

* A Friedman test was applied. Lowercase letters indicate comparisons between different time points in the same group, while uppercase letters indicate comparisons between different groups at the same time point. Labels with the same letter signify no statistically significant difference (*p* > 0.05); labels with different letters signify significant difference (*p* < 0.05).

### 3.3 Degree of repigmentation

#### 3.3.1 Mild response

Table 3 shows that in the third month, the percentage of lesions achieving a mild response was 41.4%, 62.1%, and 3.5% in the fire needle therapy, combination therapy, and topical tacrolimus ointment groups, respectively. By the sixth month, these percentages increased to 69.0%, 89.7%, and 6.9%, respectively. Significant differences were observed among the three groups at both the third‐ and sixth‐month timepoints (*p* < 0.01). Further pairwise analysis revealed that the fire needle therapy and combination therapy groups outperformed the topical tacrolimus ointment group at both the third and sixth months (*p* < 0.01). However, no substantial difference was observed between the fire needle therapy and combination therapy groups (*p* > 0.05).

**TABLE 3:** Percentage of patients with mild response.

Group	≥25% repigmentation (*n* = 29), *n*(%)	<25% repigmentation (*n* = 29), *n*(%)	*X* ^2^	*p*
After 3 months treatment			22.35	<0.01
Group A	12 (41.4%)^a^	17 (58.6%)		
Group B	18 (62.1%)^a^	11 (27.9%)		
Group C	1 (3.5%)^b^	28 (96.5%)		
After 6 months treatment			43.50	<0.01
Group A	20 (69.0%)^a^	9 (31.0%)		
Group B	26 (89.7%)^a^	3 (48.3%)		
Group C	2 (6.9%)^b^	27 (93.1%)		

*Note*: Group A, fire needle therapy; Group B, combination therapy; Group C, topical tacrolimus ointment; the overall differences among the three groups were analyzed using chi‐square test. Pairwise comparisons between the groups were adjusted using the Bonferroni method. a,b: Labels with the same letter signify no statistically significant difference; labels with different letters signify significant difference.

#### 3.3.2 Good response

As shown in Table 4, after 3 months of treatment, 50% repigmentation was observed in 1 (3.4%), 5 (17.2%), and 1 (3.4%) white patches in the fire needle therapy, combination therapy, and topical tacrolimus ointment groups, respectively. No significant differences were found among the three groups (*p* > 0.05).

**TABLE 4:** Percentage of patients with good response.

Group	≥50% repigmentation (*n* = 29), *n*(%)	<50% repigmentation (*n* = 29), *n*(%)	*X* ^2^	*p*
After 3 months treatment			4.034	0.205
Group A	1 (3.5%)^a^	28 (96.5%)		
Group B	5 (17.2%)^a^	24 (82.8%)		
Group C	1 (3.5%)^a^	28 (96.5%)		
After 6 months treatment			13.347	0.001
Group A	9 (31.0%)^a,b^	20 (69%)		
Group B	15 (51.7%)^b^	14 (48.3%)		
Group C	2 (6.9%)^a^	27 (93.1%)		

*Note*: Group A, fire needle therapy; Group B, combination therapy; Group C, topical tacrolimus ointment; the overall differences among the three groups were analyzed using Fisher's precision probability test. Pairwise comparisons between the groups were adjusted using the Bonferroni method. a,b: Labels with the same letter signify no statistically significant difference; labels with different letters signify significant difference.

After extending the treatment duration to 6 months, 15 (51.7%) white patches in the combination therapy group achieved 50% repigmentation, compared to 9 (31.0%) and 2 (6.9%) white patches in the fire needle therapy and topical tacrolimus ointment groups, respectively. A significant difference was found between the combination therapy and topical tacrolimus ointment groups (*p* < 0.01). However, no significant difference was observed between the fire needle therapy group and the other two groups (*p* > 0.05). Examples of clinical images before and after treatment are shown in Figure 7.

**FIGURE 7:**
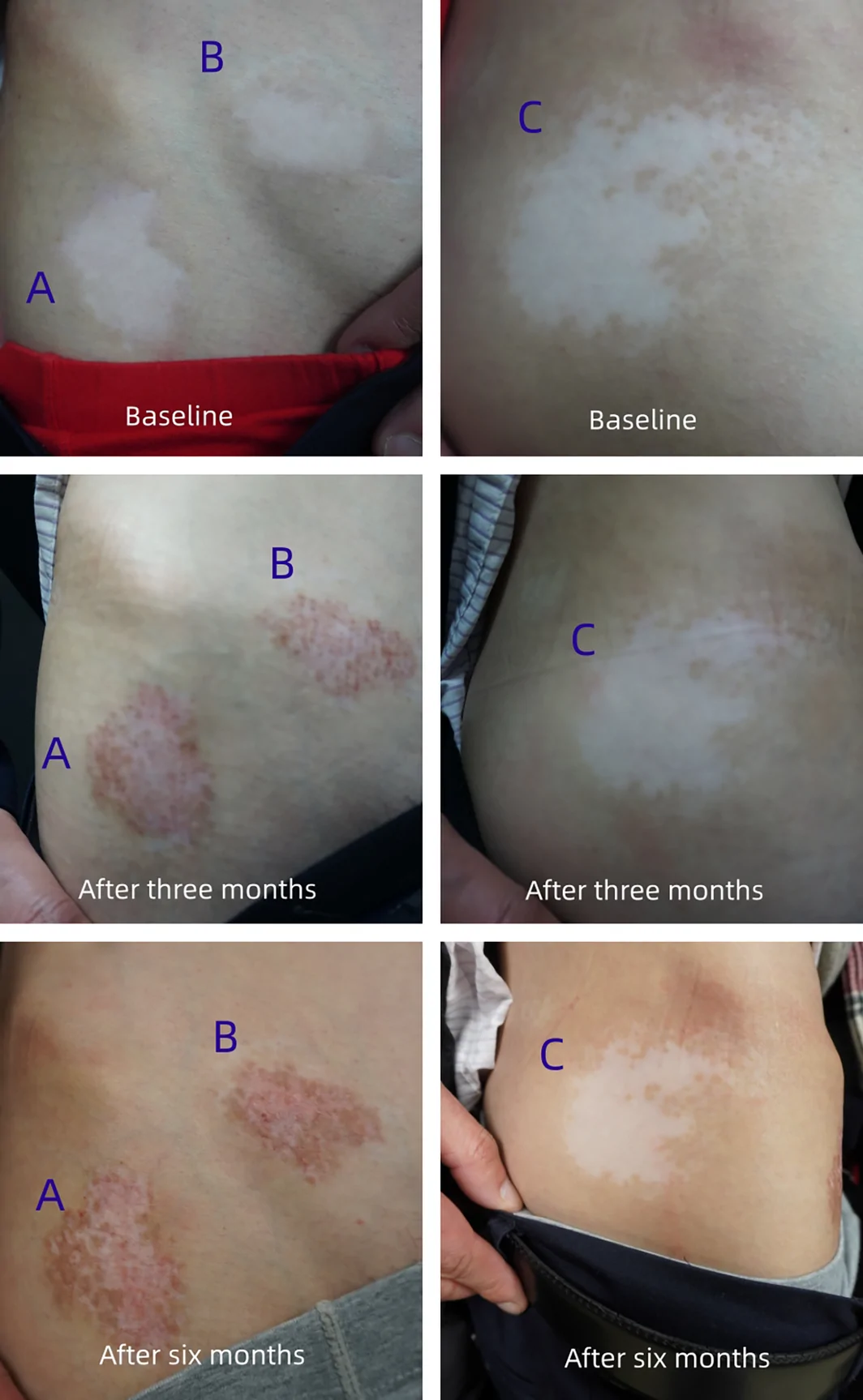
Repigmentation response in non‐segmental stable vitiligo, buttock and abdomen lesions. (A) Fire needle therapy group; (B) combination therapy group; (C) topical tacrolimus ointment group.

### 3.4 Safety

Although lidocaine cream was applied topically, all patients experienced pain during fire needle therapy. While most patients tolerated the pain, one patient withdrew from the study due to unbearable discomfort. Additionally, 16 patients developed erythema, mild itching, and scabbing following fire needle treatment, which resolved without requiring special intervention. Three patients experienced mild erythema and itching after using tacrolimus ointment.

## 4 DISCUSSION

Vitiligo remains a global therapeutic challenge, and researchers worldwide have explored various alternative treatments to enhance treatment efficacy.^25^, ^26^ Fire needle therapy, a traditional Chinese medical treatment, originated from the ancient medical text Huangdi Neijing over 2000 years ago.^27^ Over the centuries, medical practitioners have continuously refined and perfected this therapy, making it an integral part of the acupuncture system. Fire needle therapy has diverse clinical applications and can treat various skin conditions, including vitiligo, acne, flat warts, chronic eczema, neurodermatitis, prurigo nodularis, and herpes zoster.^27^

According to traditional Chinese medicine theory, fire needle therapy utilizes fire to strengthen "Yang," thereby supporting healthy "Qi" and eliminating pathogenic factors to treat vitiligo.^28^ Modern studies have also explored the mechanism of fire needling in the treatment of vitiligo. Our previous study found that micro‐injury not only triggers the migration of hair follicle melanocyte stem cells to the interfollicular epidermis but also activates them to initiate melanocyte production, assisting in refilling the vitiligo lesion.^29^ An in‐depth analysis revealed that the canonical Wnt/beta‐catenin pathway plays a pivotal role in the micro‐injury‐induced regeneration process.^29^ The study by Ye found that fire needle therapy can downregulate the chemokine CXCL12 and its receptor CXCR4, suggesting its potential role in inhibiting the infiltration of antigen‐presenting cells (APCs) and T cells into the skin, thus preventing the destruction of melanocytes.^30^ Additionally, it promotes the remodeling and regeneration of dermal collagen fibers, potentially regulating local immune cell activity, increasing the number of epidermal melanocytes, and facilitating repigmentation in vitiligo mice skin.^30^ These studies have provided additional insights into the potential mechanisms underlying the effectiveness of fire needle therapy.

This randomized, self‐controlled study investigated the therapeutic efficacy of fire needle therapy alone, 0.1% tacrolimus ointment alone, and a combination of both in patients with stable vitiligo. The results showed that the combination therapy induced repigmentation significantly faster and to a greater degree than either monotherapy. At the sixth month, nearly 90% of lesions receiving combination therapy achieved at least 25% repigmentation, and more than half achieved 50% repigmentation, which is considered a good response to treatment. Notably, fire needle monotherapy substantially outperformed tacrolimus monotherapy. By the sixth month, the fire needle group attained a 69% mild response rate, compared to just 6.9% with tacrolimus alone. These findings suggest that fire needle therapy is a more effective treatment option for stable vitiligo than topical tacrolimus ointment when used as a monotherapy.

In terms of reducing vitiligo surface area, combination therapy demonstrated superior efficacy compared to fire needle monotherapy and topical tacrolimus monotherapy, starting at months 4 and 5, respectively. Fire needle monotherapy also exhibited steady decreases in lesion size over time, with statistically significant reductions observed by the third month. In contrast, tacrolimus ointment monotherapy showed limited ability to reduce vitiligo surface area throughout the 6‐month treatment period.

Our study further substantiated the existing evidence regarding the efficacy of fire needle therapy in treating stable vitiligo. Huang's case series reported that 63.6% of vitiligo patients achieved >50% repigmentation after 3 months of fire needle therapy. Although our study did not achieve a higher repigmentation rate, it is important to note the considerable differences in study design. Huang's study employed a retrospective approach, while our study utilized a prospective randomized self‐controlled design, which minimizes selection bias and other confounding biases, enhancing the reliability of the findings. Additionally, our study excluded patients with facial vitiligo, whereas Huang's study included some patients with facial lesions, which are known to respond more robustly to therapies, potentially contributing to the higher efficacy observed in their case series.

It is noteworthy that we observed the initial appearance of irregular punctate pigment islands near the needle insertion points in the vitiligo lesions treated with fire needle therapy. These pigment islands gradually expanded, and the islands appearing at the edges of the lesions occurred earlier than those in the central areas of the lesions. Therefore, the repigmentation pattern of fire needle therapy for vitiligo is from the edges of the lesions towards the center. Furthermore, as shown in Figure 7, there is a risk of hyperpigmentation with fire needle treatment for vitiligo, where the treated areas become significantly darker than the normal skin.

Fire needle therapy's ability to induce repigmentation appears comparable to established vitiligo treatments like narrowband UVB (NBUVB). A systematic review by Bae found that 13.0% and 19.2% of patients achieved a marked response (>75% repigmentation) after 3 and 6 months of NBUVB treatment, respectively.^31^ Further rigorous studies directly comparing fire needle therapy against phototherapy modalities like NBUVB would help definitively establish the efficacy of fire needling relative to first‐line treatments. Head‐to‐head randomized controlled trials comparing these interventions are warranted.

Our study demonstrated that combining fire needle therapy with 0.1% tacrolimus ointment resulted in greater repigmentation than either monotherapy, aligning with Yang et al.'s findings confirming the superiority of combination therapy.^32^ In their study, the percentage of repigmentation was significantly higher in the group receiving combined electrocautery needling and excimer lamp treatment versus monotherapy groups. In another study, the combination of CO_2_ fractional laser and tacrolimus was employed for the treatment of vitiligo. The findings revealed that 90% of the patients demonstrated a good response, surpassing the outcomes of our research. Nevertheless, it is important to note that the aforementioned study had a limited sample size of only 10 participants.^33^ Fire needle therapy inserts red‐hot needles into the lesion, facilitating medication penetration and absorption through increased skin temperature and micro‐trauma.^29^ Thus, when combined with topical medication, the enhanced transdermal drug delivery facilitated by fire needle therapy may contribute to the higher efficacy of combination therapy compared to either therapy alone.

To minimize discomfort, lidocaine cream was applied prior to fire needling. Overall, this treatment was well‐tolerated, with only one patient withdrawing due to pain and no other serious AEs. Although a previous study noted potential keloid formation following fire needling for vitiligo,^18^ careful patient selection excluding those prone to scarring prevented these complications in our study. Our results suggest that fire needle therapy can be a safe approach for vitiligo when proper precautions are taken.

This study has some limitations worth noting. First, our data was collected from a single center, so the results may not be widely generalizable due to potential regional differences in patient populations. Additionally, although we performed sample size calculations, the actual repigmentation responses in all three groups were lower than estimated, and we had 6 patients drop out, both of which reduced our statistical power.

## 5 CONCLUSIONS

This study provides promising evidence supporting the use of fire needle therapy, alone or in combination with topical agents like tacrolimus ointment, for stimulating repigmentation in non‐segmental stable vitiligo. Given the limitations of existing treatment options, our findings suggest that fire needle therapy warrants consideration as an alternative therapeutic strategy. Advantages include its non‐pharmacological nature, low risk profile, and potential for achieving continual repigmentation with repeated treatments over time. Longer‐term studies with larger sample sizes are imperative to further characterize the efficacy and safety of this traditional Chinese medicine technique. Clinical trials comparing fire needle therapy against first‐line treatments like phototherapy would also help establish its place within the vitiligo treatment paradigm.

## AUTHOR CONTRIBUTIONS

Y.W., Q.D., M.L., and Z.L. conceived the study. F.H., R.J., W.X., Q.H., Q.H., and Q.X., assist the clinical recruitment process. Y.W, and F.H., analyzed the data. Y.W., wrote the main manuscript text. Z.L., Q.D., and M.L., revised the manuscript. All authors contributed to the article and approved the submitted version.

## FUNDING INFORMATION

This work was supported by the National Natural Science Foundation of China (82374298) and Natural Science Foundation of Chongqing (2020ZY3807, 2020ZY013906).

## CONFLICT OF INTEREST STATEMENT

The authors state that there are no known financial conflicts of interest or personal relationships that could have potentially influenced the findings presented in this paper.

## Data Availability

The data that support the findings of this study are available from the corresponding author upon reasonable request.
